# IL-10 Dampens the Th1 and Tc Activation through Modulating DC Functions in BCG Vaccination

**DOI:** 10.1155/2019/8616154

**Published:** 2019-06-12

**Authors:** Hui Xu, Yanjuan Jia, Yonghong Li, Chaojun Wei, Wanxia Wang, Rui Guo, Jing Jia, Yu Wu, Zhenhao Li, Zhenhong Wei, Xiaoming Qi, Yuanting Li, Xiaoling Gao

**Affiliations:** The Institute of Clinical Research and Translational Medicine, Gansu Provincial Hospital, China

## Abstract

BCG, the only registered vaccine against *Mycobacterial Tuberculosis* (TB) infection, has been questioned for its protective efficacy for decades. Although lots of efforts were made to improve the BCG antigenicity, few studies were devoted to understand the role of host factors in the variability of the BCG protection. Using the IL-10KO mice and pulmonary tuberculosis infection model, we have addressed the role of IL-10 in the BCG vaccination efficacy. The data showed that IL-10-deficient dendritic cells (DCs) could promote the immune responses through upregulation of the surface costimulatory molecule expression and play an orchestra role through activating CD4^+^T cell. IL-10-deficient mice had higher IFN *γ*, TNF *α*, and IL-6 production after BCG vaccination, which was consistent with the higher proportion of IFN *γ*^+^CD3^+^, IFN *γ*^+^CD4^+^, and IFN *γ*^+^CD8^+^ T cells in the spleen. Particularly, the BCG-vaccinated IL-10KO mice showed less inflammation after TB challenge compared to WT mice, which was supported by the promoted Th1 and Tc, as well as the downregulated Treg responses in IL-10 deficiency. In a conclusion, we demonstrated the negative relationship between Th1/Tc responses with IL-10 production. IL-10 deficiency restored the type 1 immune response through DC activation, which provided better protection against TB infection. Hence, our study offers the first experimental evidence that, contrary to the modulation of BCG, host immunity plays a critical role in the BCG protective efficacy against TB.

## 1. Introduction

*Mycobacterial Tuberculosis* (MTB) remains a detrimental contagious disease responsible for about 3 billion infection. According to the World Health Organization's (WHO) statistics, its scourge claims close to 1.6 million deaths worldwide each year (World Health Organization. Available at: http://www.who.int/mediacentre/factsheets/fs104/en/) aided by an endemic of multiple drug resistant strains, poor adherence to a long duration of therapy, HIV infection-induced immune compromise, and social and economic public health constraints [1].

The Bacillus Calmette-Guerin (BCG) is the only approved antituberculous vaccine developed through the serial in vitro passage of *M. bovis* until it becomes unpathogenic [2], but its efficacy had been questioned for decades. Even it can confer reliable protection in children, it is inconsistent in limiting adult TB infection [3]. More interestingly, BCG decreases overall morbidity and mortality to other infectious diseases, which suggests that BCG offers the protective immune response to non-TB pathogens. Furthermore, BCG possesses potent immunostimulatory properties in the treatment of bladder cancer, multiple sclerosis, and type 1 diabetes [2, 4–6]. Given its potentials, beyond doubt, BCG is still the successful, affordable, and promising vaccine, especially in a resource-limited area.

New anti-TB vaccine development focuses on the improving BCG by enhancing its immunogenicity or applying the prime-boost strategies to amplify the protective response [7–11]. However, the suppressive host microenvironment would circumvent the protective immune responses. Before we move forward with the development of a new or an improved TB vaccine to combat this pathogen, it is vital for better exploring the host limitations on BCG-induced protection.

Currently, there is limited efforts on defining the host factors in modulating the efficacy of BCG. The ability of host to control TB infection is largely dependent on the antigen-presenting cells such as macrophage and dendritic cells (DCs) to initial adaptive immune responses. Even both macrophage and DCs can be infected by TB, macrophage is rather equipped to control internalized TB through the NO production, while the TB can hijack the macrophage to escape the immune surveillance. On the other hand, DCs are best-known as the main participants in mounting the effective T cell-mediated immune responses against TB [12]. As a result, DCs are considered as a target for novel TB vaccine strategies [8]. Since DCs are a heterogeneous population, different DC subtypes contribute differently on the activation and polarization of TB-specific T cells. The phenotypic and functional alterations in DCs have been reported in individuals with TB infection. The type-1 polarized DCs (DC1s) induce Th1 responses and CTLs in TB infection [13]. Furthermore, regulatory DCs (DCreg) possess the suppressive potentials by secreting a number of soluble factors, e.g., PGE2, IL-10, and NO, which are capable to inhibit T cell responses [14–16]. Although we and others have previously shown the critical role of DCs in protective immunity in TB infection, the power of DCs in vaccine development has yet to be realized [17–19].

Cytokine interleukin-10 (IL-10) has been implicated in the pathogenesis of TB [20]. IL-10 is an important immunoregulatory cytokine mainly produced by macrophages, DC, monocytes, T cells, et al. [21]. Turner et al. demonstrated that increased susceptibility of reactivation of latent infection was strongly related to the expression of IL-10 during the chronic or latent phase of the infection [22]. IL-10 helps TB persistence in human by blocking phagosome maturation in macrophages [23]. The ability of IL-10 to downregulate immune responses and the fact that IL-10 can be detected in tuberculosis patients have led us to investigate whether IL-10 plays a role in BCG vaccination efficacy [24, 25].

In current study, we implied IL-10KO mice to explore the potential role of IL-10 in the BCG vaccination efficacy. Our data supported that DC activation was hampered after BCG vaccination due to the IL-10 production. IL-10 prevented the development the protective Th1 immune response which may be associated with the increased Treg activity.

## 2. Materials and Methods

### 2.1. Mice and Culture Medium

The animal was handled in compliance with the Guidelines for Animal Use set by the Ethic Committee on animal care and use. C57BL/6 female mice (6-8 weeks) and IL-10KO mice (Nanjing Junke Bioengineering Co., China) were housed for 2 weeks before the study. This study was approved by the Ethics Committee of Gansu Provincial Hospital. RPMI 1640 medium (Biological Industries, Israel) supplemented with 10% HI-FBS (heat-inactivated fetal bovine serum), 1% L-glutamine, 25 *μ*L/mL gentamycin, and 5 × 10^−5^ M_2_ was used as complete medium for cell culture. PBS was used as the control.

### 2.2. Organism and Model of BCG Vaccination and Challenge (Figure 1)

The BCG strain was made by Wuhan Biological Products Research Institute Co. LTD. For expansion of the vaccine, the protocol was applied based on previous study [12]. Briefly, BCG was grown in the Middlebrook's 7H9 broth (BD Difco, USA) containing 0.2% (*v*/*v*) glycerol and 0.05% (*v*/*v*) Tween-80 and supplied with 10% (*v*/*v*) Middlebrook ADC enrichment (Difco) for 21days. The stock of BCG was detected for the numbers of bacilli before it was stored at -80°C before use. The colony-forming units (CFUs) were measured by plating diluted culture on plates of Middlebrook 7H11 agar (Difco) containing 0.5% (*v*/*v*) glycerol and supplied with Middlebrook OADC enrichment (Difco). BCG was put at 65°C for 1 hour for inactivation, which led to complete killing of BCG confirmed by viability testing (HK BCG). Mice were immunized intravenously with 5 × 10^5^ CFU BCG in 200 *μ*L sterile protein-free PBS and sacrificed at day 21 after immunization as previously described [26, 27]. Spleens were aseptically isolated and digested in 1.5 mg/mL collagenase D (Sigma, USA) at 37° for 30 min. The single cells were prepared after cell suspension went through cell strainer (70 *μ*m). DC surface marker expression was collected by Aria II flow cytometer (BD, CA, USA) and analyzed using FlowJo software. Vaccinated mice were challenged intranasally (i.n.) with 5 × 10^6^ CFUs of BCG. Mice were euthanized 21 d later and analyzed for immune responses.

### 2.3. Lung, Spleen, and Local Lymph Node Cell Isolation

Single cells isolated from the lungs, spleen, and draining lymph node (dLN) were set for bulk culture and flow cytometry analysis as described [28, 29]. Briefly, spleens were cut into small pieces and digested in 1.5 mg/mL collagenase D in RPMI 1640 for 30 min at 37°C. The cell suspension was filtered through cell strainer (70 *μ*m), and RBCs were removed by ammonium-chloride-potassium (ACK) lysis buffer (150 mmol/L NH_4_Cl, 10 mmol/L KHCO_3_, and 0.1 mmol/L EDTA). Lung tissues were harvested aseptically and digested in 2 mg/mL collagenase XI (Sigma, USA) in RPMI 1640 for 1 h at 37°C. After digestion, 35% (volume/volume) Percoll (Pharmacia, USA) was used to collect lung single cells. And ACK lysis buffer was used to remove red blood cells (RBCs). For dLN mononuclear cell isolation, the dLNs were homogenized in 3 mL RPMI 1640 and RBCs were removed by ACK lysis buffer. All of the cells were washed and resuspended in complete RPMI-1640 medium. Single-cell suspensions were cultured in 48-well plates at a concentration of 7.5 × 10^6^ (spleen) and 5 × 10^6^ (lungs and dLNs) cells/well with HK BCG (5 × 10^5^ CFU/mL). The supernatants were collected from the cell cultures after 3 d and assayed for IFN *γ*, IL-4, IL-6, and TNF *α* production by Enzyme-Linked Immunosorbent Assay (ELISA) using antibodies purchased from eBioscience or BD Biosciences [12].

### 2.4. Isolation of CD4^+^T Cell and DC Coculture

Spleen CD4^+^T cells were isolated from WT mice after BCG vaccination as we previously described [30]. DCs were purified from the spleens of WT and IL-10KO BCG-vaccinated mice by flow cytometric sorting. DCs (7.5 × 10^5^ cells/mL) from WT or IL-10KO BCG-vaccinated mice were cocultured with CD4^+^T cells (7.5 × 10^6^ cells/mL) in 96-well plates in the presence of HK-BCG (5 × 10^4^ CFU/mL) in 200 *μ*L complete RPMI medium. For testing IFN *γ* and IL-4 production, the supernatants were collected at 72 h and tested by ELISA.

### 2.5. Analysis of Lung Pathology

The lungs of WT or IL-10KO mice after BCG challenge were collected and fixed in 10% buffered formalin. Lung tissues were embedded, sectioned, and stained by H&E as described [26, 31]. Infiltrating inflammatory cells were identified based on cellular morphology and characteristics. Slides were examined for pathological changes by 2 independent pathologists using a light microscopy.

### 2.6. Flow Cytometric Analysis

The intracellular IFN *γ*- or Foxp3-positive T cells were analyzed by intracellular cytokine staining as described previously [28]. Briefly, the cells were stimulated with phorbolmyfismte acetate (PMA; 50 ng/mL) and ionomycin (1 g/mL) for 2 hours, then brefeldin A was added to the culture, and the cells were cultured for another 4 h to accumulate cytokine intracellularly. The cells were collected and stained with anti-CD3*_Ɛ_*-fluorescein isothiocyanate (FITC), anti-CD4-phycoerythrin (PE) mAbs, and anti-CD8-PerCy5.5 (BD, CA, USA). After being fixed and washed in permeabilization buffer twice, cells were stained with anti-IFN *γ* allophycocyanin (APC). For Treg detection, cells were stained with FITC-anti-CD3*_Ɛ_*, PerCy5.5 anti-CD4, APC anti-CD25, and PE-Foxp3. All of the sample data were collected using an Aria II flow cytometer (BD, CA, USA) and analyzed using FlowJo software.

### 2.7. Statistical Analysis

Statistical analysis of the data was performed using analysis of variance (ANOVA) and *t* tests (GraphPad Prism software, version 5.0 (GraphPad Software, La Jolla, CA, USA)), and values of *p* < 0.05 were considered significantly. Data are presented as mean ± standard deviation (SD). The presented data were collected from four to five mice of each group. All the experiments were repeated at least three times with similar results.

## 3. Results

### 3.1. Less Inflammation after IL-10KO in TB Challenge

**Figure 2:**
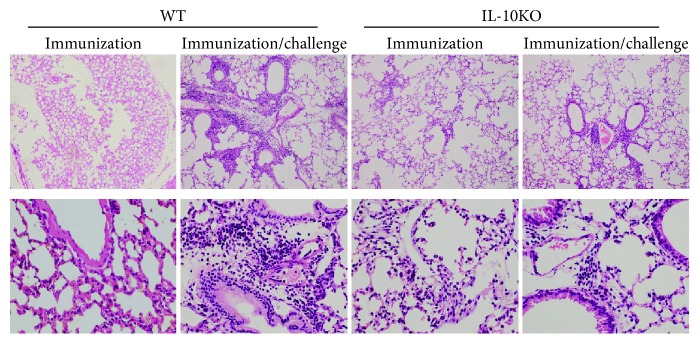
The pathological changes in vaccinated IL-10KO and WT mice following intranasally (i.n.) challenge infection after BCG vaccination. Pretreated mice were challenged intranasally with 5 × 10^6^ CFUs BCG and analyzed for histopathological changes in the lungs at day 21 postchallenge infection. Lung tissue sections (6 *μ*m) were stained with H&E for inflammation. (a) Low magnification (×10), (b) High magnification (×40). The representative of three independent experiments (*n* = 5) with similar results was shown.

To further assess the role of IL-10 in BCG vaccination efficacy, we challenged the BCG-vaccinated mice intranasally with high dose of BCG (5 × 10^6^) and detected inflammation in the lungs. As shown in Figure 2, IL-10KO mice pretreated with BCG showed mild infiltrated inflammation in the lungs against challenge. In contrast, the WT mice immunized and challenged with BCG have much more severe pathological inflammatory reactions. Interestingly, both WT and IL-10KO mice have very mild inflammatory cells in their lungs after BCG immunization without challenge. The data suggested that IL-10KO deficiency can dramatically modulate the pathological inflammatory responses in the local tissues [12].

### 3.2. IL-10 Deficiency Enhances Th1 and Cytotoxic T Cells after BCG Challenge Intranasally

**Figure 3:**
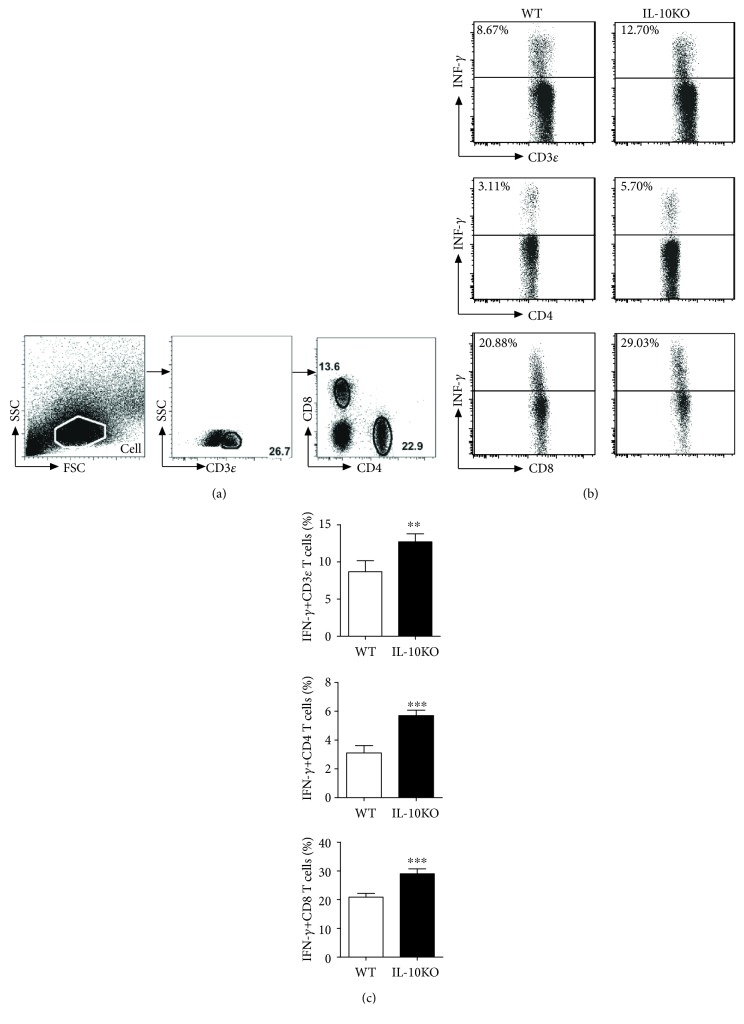
IL-10KO mice showed higher IFN *γ* production after BCG challenge in the lungs. The mice were treated as described in the legend of Figure 4. The lungs were digested as described in Materials and Methods. The intracellular IFN *γ*-positive T cells were analyzed by intracellular cytokine staining as described previously [29]. (a) Gating strategy: firstly, the major cells were gated for further gated based on the CD3*_Ɛ_* expression. Then, the CD3*_Ɛ_*^+^ cells were gated on the CD4 and CD8 expression for CD4^+^ and CD8^+^ T cells. (b) The figures from FAC analysis for the IFN-*γ*^+^ expression were shown. (c) Summary of the percentage of the IFN-*γ*^+^CD3*_Ɛ_*^+^, CD4^+^, and CD8^+^ T cells in the lungs, and Student's *t* test was used for analysis. The representative of three independent experiments (*n* = 5) with similar results was shown. ^∗∗^*p* < 0.01, ^∗∗∗^*p* < 0.001.

**Figure 4:**
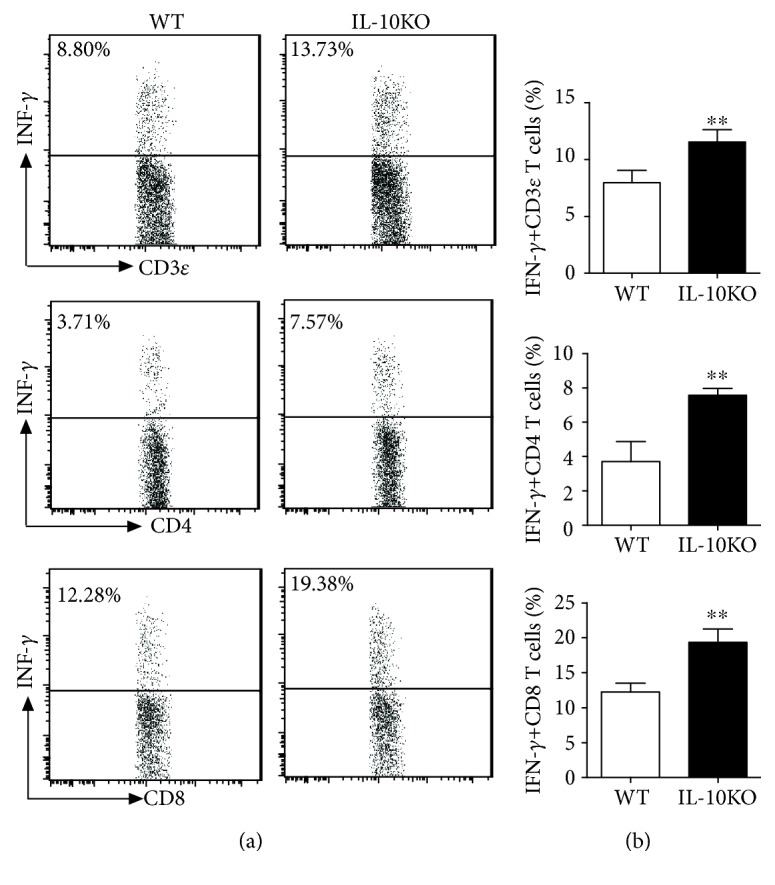
IL-10KO mice showed higher IFN *γ* production after BCG challenge in dLN. The mice were treated as described in the legend. The single cells from dLN were stained, and the intracellular IFN *γ*-positive T cells were analyzed by intracellular cytokine staining as described previously [29]. (a) Representative figures from FAC analysis were shown. (b) Summary of percentage of the IFN *γ*^+^CD3*_Ɛ_*^+^, CD4^+^, and CD8^+^ T cells in dLN. Data are shown as the representative of three independent experiments with similar results (*n* = 5) and Student's *t* test was used for analysis. ^∗∗^*p* < 0.01.

To examine the immune responses in BCG-challenged IL-10KO mice and WT mice, we performed the intracellular cytokine staining for T cells from the lungs and draining lymph nodes collected from mice after BCG challenge. As shown in Figures 3 and 4, IL-10-deficient mice showed higher IFN *γ* production in CD3*_Ɛ_*^+^T cells, which was consistent with the protective responses observed in lung pathogenesis changes, same to IFN *γ*^+^ CD4^+^and IFN *γ*^+^ CD8^+^T cells in both lungs and dLN in IL-10KO mice. Taken together, the results suggest that IL-10 blocks the Th1 responses, as well as cytotoxic T cell development in mycobacterial infection. IL-10 production might be partially responsible for the ineffective BCG vaccination.

### 3.3. Th1 and Tc Responses Were Restricted by IL-10 Production in BCG Vaccination

**Figure 5:**
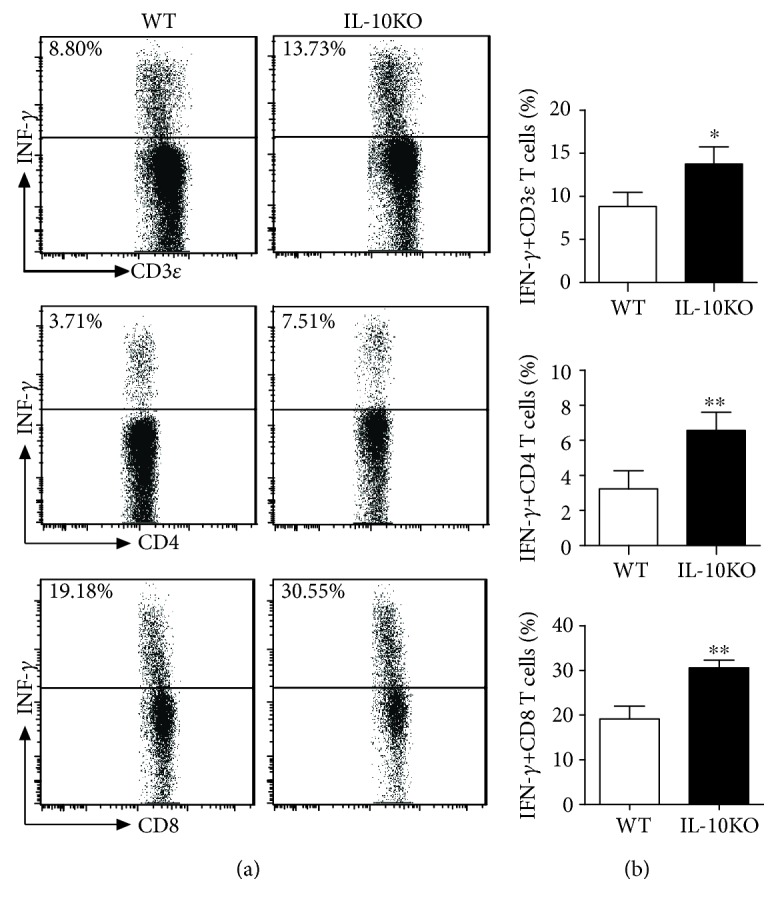
IL-10KO mice had higher IFN *γ* production after BCG vaccination in the spleen. The spleen was digested with enzyme as described in Materials and Methods. The cells were stained with FITC anti-CD3*_Ɛ_*, PE anti-CD4, PerCy5.5 anti-CD8, and APC anti-IFN *γ*. The intracellular IFN *γ*-positive cells were analyzed by intracellular cytokine staining when cells were gated on CD3*_Ɛ_*, CD4, and CD8 as described previously [29]. (a) Representative figures from FAC analysis were shown. (b) Summary of percentage of the IFN *γ*^+^CD3*_Ɛ_*^+^, CD4^+^, and CD8^+^ T cells in the spleen, and Student's *t* test was used for analysis. ^∗^*p* < 0.05, ^∗∗^*p* < 0.01. The representative of three independent experiments (*n* = 5) with similar results was shown.

It is well known that Th1 and Tc were critical for TB infection control. To further explore the role of IL-10 in Th1 and Tc development during the BCG vaccination, the T cell subsets were then examined in the IL-10KO and WT mice after BCG vaccination. The cells isolated from the spleen were analyzed by multicolor staining. As shown in Figure 5, IL-10KO promoted dominant Th1 (IFN *γ*^+^CD4^+^T cells) production and Tc production (IFN *γ*^+^CD8^+^T cells). It suggested that, without IL-10, BCG vaccination could induce better Th1 and Tc immune responses that might offer better protection against TB infection.

### 3.4. IL-10KO Mice Showed Significantly Higher Protective Cytokine Production after BCG Vaccination in the Spleen

**Figure 6:**
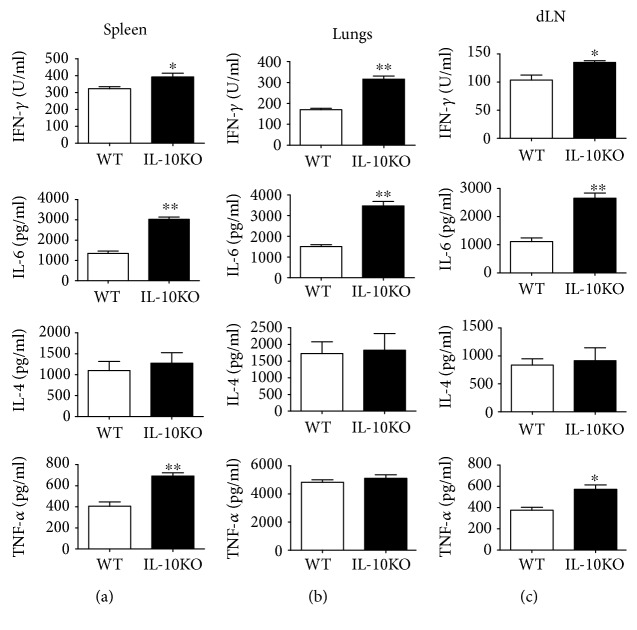
Cytokine levels after BCG vaccination in IL-10KO mice compared to WT mice. IL-10KO and WT mice were immunized with BCG as described in Materials and Methods. At d 21 after vaccination, the single cells prepared from the spleen, lungs, and dLN were cultured for 72 h stimulated with HK-BCG (5 × 10^5^ CFUs/mL) before supernatant collection. The cytokines were measured by the ELISA. Data were summarized and representative of three independent experiments with similar results were shown. ^∗^*p* < 0.05, ^∗∗^*p* < 0.01.

To further explore the systemic immune responses in WT vs. IL-10KO mice after BCG immunization, the spleen, lungs, and draining lymph nodes were collected aseptically and the bulk culture of splenocytes and the cells isolated from draining LN and lung tissues were used to detect the cytokine production in the supernatant in both IL-10KO and WT mice after BCG vaccination. As shown in Figure 6, IFN *γ* and IL-6 levels showed significantly higher in the IL-10KO mice compared to WT mice, but comparable levels of IL-4 were found in the spleen, lungs, and dLN in both types of mice. Rather surprisingly, the TNF *α* levels of IL-10KO mice were higher than those in WT mice in the spleen and dLN but not in the lungs. The data suggest that IL-10 inhibited the protective IFN *γ* and TNF *α* expression, as well as IL-6 production, even with the comparable TNF *α* expression in the lungs from both types of mice.

### 3.5. IL-10 Inhibits the T Cell Activation by DCs in BCG Vaccination

**Figure 7:**
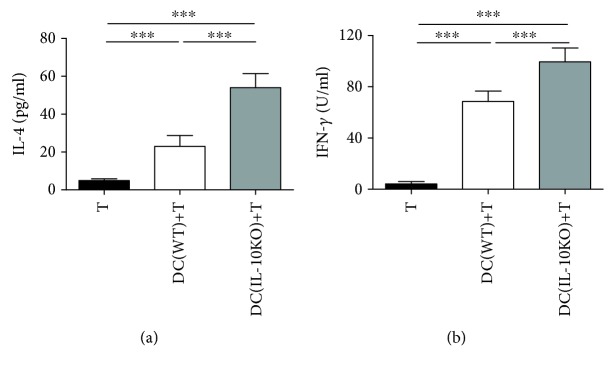
Cytokine production from DC-CD4^+^T cell coculture system. Spleen DCs (7.5 × 10^5^ cells/mL) from WT and IL-10KO BCG-vaccinated mice were cocultured with BCG-educated CD4^+^T cells (7.5 × 10^6^ cells/mL) as described in Materials and Methods. Cell supernatants were collected at 72 h for IFN *γ* and IL-4 detection by ELISA. Data were summarized as mean ± SD (*n* = 5) and representative of three independent experiments with similar results were shown. ^∗∗^*p* < 0.01, ^∗∗∗^*p* < 0.001.

To detect the role of IL-10 in the DC function of directing the T cell differentiation, the spleen DCs from WT and IL-10KO mice after BCG vaccination were purified by the FACs. The CD4^+^T cells were isolated from the spleen of BCG-vaccinated WT mice. The DCs were cocultured with BCG-boosted CD4^+^T cells. The IL-4 and IFN *γ* production in the culture supernatants were detected. As shown in Figure 7, the DCs from IL-10KO mice initiated higher IL-4 and INF *γ* production by CD4^+^T cells than those from WT mice (*p* < 0.01). The results demonstrated that IL-10 signaling may contribute to the DC dysfunction in BCG vaccination.

### 3.6. IL-10 Prevents the Activation of DC after BCG Vaccination

The functions of DC in initiating immune responses largely depend on their expression of costimulatory molecules on their surface. To detect the role of IL-10 production in modulating DC functions in BCG vaccination, FACs were applied to detect the surface marker expression on the spleen DCs after BCG immunization. As shown in Table 1, there is no much jump between WT and IL-10KO mice regarding the CD80, CD86, and CD40 expression before the vaccination. However, after BCG immunization, the spleen DCs from IL-10-deficient mice expressed higher CD80 (89.79% vs. 58.32%), CD86 (32.66% vs. 25.73%), and CD40 (20.95% vs. 15.38%) molecules in comparison with the DCs from WT mice. It suggested that BCG vaccination can activate DCs which were suppressed by the IL-10 production.

### 3.7. IL-10KO Mice Showed Reduced Tregs after BCG Vaccination

**Figure 8:**
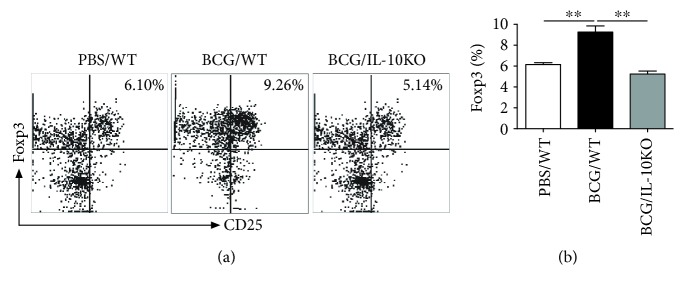
Lung Foxp3^+^ Treg cells were analyzed after BCG challenged in IL-10KO and WT mice. The mice were treated as described in the legend of Figure 7. Lung single cells were stained with FITC anti-CD3*_Ɛ_*, PerCy5.5 anti-CD4, APC anti-CD25, and PE-Foxp3. The Foxp3 expression was analyzed by flow cytometry when cells were gated on CD3*_Ɛ_*^+^CD4^+^T cells. (a) The representative pictures of Foxp3^+^CD3*_Ɛ_*^+^CD4^+^CD25^+^Tregs were shown. (b) Summary of the frequency of Foxp3^+^CD25^+^CD4^+^T cells in total T cells in the lungs. Data are shown as mean ± SD (*n* = 5) and are representative of three independent experiments with similar results. ^∗∗^*p* < 0.01.

The role of regulatory T cells (Tregs), especially Foxp3^+^ Tregs, was investigated after TB challenge infection. It was shown that the increased Treg was related to the pathogenesis in TB infection [32]. Treg analysis in the lung tissues by FACs showed less Foxp3^+^CD25^+^CD4^+^ T cells in the lungs of IL-10KO mice after BCG challenge (Figure 8). Collectively, the results demonstrated the increased IFN *γ* expression without IL-10 but reduced Treg responses following BCG challenge.

## 4. Discussion

The protective efficacy of BCG is variable against TB infection. Some studies were devoted to BCG engineering for increasing its antigenicity to drive a desirable immune response against TB [33, 34]. The potential to modulate host immunity is critical for the success of a vaccine. The host genetic heterogeneity is partially responsible for the BCG failure that the host factors might be targeted to enhance the potency of this vaccine. In this study, we aimed to investigate the roles of host factor IL-10 in BCG vaccination efficacy. The data supported that BCG vaccination in IL-10-deficient host can boost the DC activation through upregulation of the surface molecule expression. In vivo BCG immunization in IL-10KO mice promotes IFN *γ* production, which was consistent with the protective effects after BCG challenge. The mild lung pathologic changes after BCG vaccination/challenge supported that IL-10 deficiency resulted in higher protective responses. In clinical practice, an increased susceptibility toward the development of progressive tuberculosis in humans with increased IL-10 levels was reported [35]. Our study explored the molecular mechanisms of the notorious role of IL-10 in the BCG vaccination.

The ability of the host to control TB infection depends on recruited Th1 cells to release and activate the killing mechanisms of the infected cells. In the current study, to assess Th1-polarizing capacity after comparing the immunoregulatory cytokine IL-6, IFN *γ*, IL-4, and TNF *α* production, we found that IL-10KO mice produced more IFN *γ* and TNF *α* compared to the WT mice. The predisposition of IFN *γ* gene-deficient mice to TB and the therapeutic role of IFN *γ* support an essential role for this cytokine in the protective immune response against TB. Additionally, the clinical application of anti-TNF *α* leads to reactivation of TB infection or patients succumbed to the TB disease. It is known that, after TB infection, activated type 1 T cells, particularly CD4^+^T cells, are the most abundant cellular source of IFN *γ*. Consistent with the data of bulk culture, there was a much less frequency of IFN *γ* producing CD3*_Ɛ_*^+^, CD4^+^, and CD8^+^T cells in the spleen in WT mice than those in IL-10 KO mice.

The role of IL-10 in the immune response during TB infection had been studied previously. Several papers had shown that IL-10 is mainly produced by hematopoietic cells and play an important role in suppressing macrophage and dendritic cell (DC) functions. Thus, IL-10 is responsible for the ability of TB to evade immune responses [36]. Another study investigated the role of IL-10 in BCG efficacy too. The authors showed that IL-10 signaling deficiency during BCG vaccination enhanced Th1 and Th17 responses that improved the protection against TB infection [37]. Our study focuses on and detects the functions of dendritic cells, the orchestra of immune response, and found that IL-10 hinders the activation of DCs that delays the fully activation of T cell responses. Further, our study firstly showed that IL-10 promoted the Treg development. We found that the frequency of regulatory T cells increased in the IL-10-competent mice, while decreased in IL-10 compromised ones. Our study indicated that the host factors, especially the dendritic cell function, maybe of importance in understanding the efficacy of vaccine. It is a great benefit to develop a proper adjuvant for the efficacious vaccination strategy.

It is believed that BCG “educated” DCs interact with CD4^+^T cells that they reciprocally affect each other's activation. Interestingly, we found that IL-10-deficient DCs can induce both higher IL-4 and IFN *γ* production by the CD4^+^T cell in vitro coculture system. It is well known that Th2 responses promote TB progression by inhibiting Th1 responses. Given an activation of both Th1 and Th2 T cells in the DC-CD4^+^T cell coculture system (Figure 7), we examined the impact of IL-10 deficiency on the T cell activation by using intracellular cytokine staining after BCG immunization (Figure 5). To this end, the total T cell, CD4^+^, and CD8^+^T cell subsets isolated from IL-10KO mice showed enhanced IFN *γ* production in the spleen. It indicated that in vivo host immunity lean to Th1 responses without IL-10 in BCG vaccination. In line with this finding, the IL-10-deficient mice did not show any Th2 response after BCG vaccination/challenge. We speculated that both Th2 and Th1 cells were released without IL-10 production, but the extent and level of Th2 are much less than Th1 in vivo that IL-4 production is comparable in IL-10KO and WT mice. Another reason for the inconsistent IL-4 production between in vivo T cell responses and in vitro coculture study is that T cells in the in vitro coculture system were capable to produce IL-10 due to its isolation from WT mice, while in vivo BCG vaccination and challenge were conducted in the IL-10KO mice that T cell responses in the background of IL-10 deficiency.

After BCG challenge in immunized mice, IL-10KO mice had much higher Th1 responses in both lungs (Figure 3) and dLN (Figure 4). It is consistent with the lung inflammation detected by the professional pathologists that much less proinflammatory response in the IL-10KO mice compared to that in WT mice. It is keeping with Shaler et al. who showed that IL-10 deficiency can restore the Th1 response in a granuloma using IL-10KO mice [38].

It is noteworthy that an attenuated strain of mycobacteria (BCG, *M. bovis*), but not virulent *Mtb*, was applied in our current study that the same antigen was used for both vaccination and challenge. However, studies worked on the comparison of BCG and virulent strain of *Mtb* in mice showed that the overall kinetics and cellular response were comparable, same to the protective roles of IFN *γ* and Th1 responses in either BCG or *Mtb* exposure in mice [39–41]. Despite all these described similarities, we must caution to explain our findings in the clinical setting since *Mtb* is significantly more virulent compared to BCG.

Given that BCG vaccination efficacy is far from satisfied and much efforts focuses on the antigen modification, our current findings provide a new strategy for better vaccine adjuvant development, as well as a plausible mechanism to explain the heterogeneous outcomes after BCG vaccination in different populations.

## Figures and Tables

**Figure 1:**
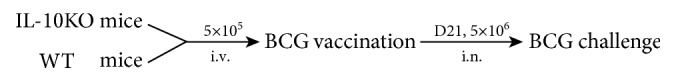
The chart of BCG vaccination and challenge to establish the BCG vaccine/challenge mouse model. The IL-10KO and WT mice were housed for 2 weeks after arriving. For vaccination, the mice were intravenously injected with 5 × 10^5^ CFU BCG in 200 *μ*L PBS; the control group was given PBS only. For challenge, at D21 after vaccination, the mice were infected intranasally with 5 × 10^6^ CFU BCG. The immune responses were detected after 21 days.

**Table 1:** The surface molecular expression on DC from WT/IL-10KO mice ($\bar{\text{x}}\pm \text{SD}$).

	BCG immunization		PBS (naïve)	
	WT (*n* = 5)	IL-10KO (*n* = 5)	WT (*n* = 5)	IL-10KO (*n* = 5)
CD80 (%)	58.32 ± 2.50	89.79 ± 9.20^∗^	22.14 ± 3.12	23.47 ± 2.90
CD86 (%)	25.70 ± 4.47	32.66 ± 3.16^∗^	11.06 ± 2.20	10.66 ± 2.33
CD40 (%)	15.38 ± 3.54	20.95 ± 3.03^∗^	7.17 ± 3.54	5.32 ± 1.10

^∗^ *p* < 0.05, compared to the WT mice.

## Data Availability

The scientific and statistical data used to support the findings of this study are included within the article. Requests for access to these data should be addressed to Xiaoling Gao at Gaoxl008@hotmail.com.
